# Discontinuation of proton pump inhibitors during assessment of chromogranin A levels in patients with neuroendocrine tumours

**DOI:** 10.1038/bjc.2011.380

**Published:** 2011-10-11

**Authors:** C M Korse, M Muller, B G Taal

**Affiliations:** 1 Department of Clinical Chemistry, The Netherlands Cancer Institute – Antoni van Leeuwenhoek Hospital, PO Box 90203, 1006 BE Amsterdam, The Netherlands; 2 Department of Medical Oncology, The Netherlands Cancer Institute – Antoni van Leeuwenhoek Hospital, Amsterdam, The Netherlands

**Keywords:** neuroendocrine tumours, chromogranin A, proton pump inhibitors, H_2_-receptor antagonists

## Abstract

**Background:**

The aim of this prospective study was to examine whether discontinuation of proton pump inhibitors (PPIs) or replacement by H_2_-receptor antagonists (H2RA) resulted in a decrease of chromogranin A (CgA) levels in 196 patients with well-differentiated neuroendocrine tumours (NETs).

**Methods:**

Patients with an unexpectedly high CgA level not connected with NET disease discontinued PPIs, or used H2RA instead; 2 weeks later CgA level was measured again.

**Results:**

In all, 19 out of 196 (10%) patients showed unexpected elevated CgA levels, they all used PPI. In 11 out of 19 patients with no evidence of the disease, median CgA decreased from 390 *μ*g l^−1^ during PPI treatment to 56 *μ*g l^−1^ after discontinuation (*P*=0.003). In 8 out of 19 patients with stable disease, median CgA decreased from 618 to 318 *μ*g l^–1^ (*P*=0.012). In 12 out of 19 patients who ceased all acid inhibition, CgA levels decreased by 82%, while in the seven patients who replaced PPI by H2RA, CgA decreased by 77% (*P*=0.967).

**Conclusion:**

Proton pump inhibitor use causes falsely elevated CgA levels in patients with NET. We recommend to stop, or replace PPI by H2RA, to obtain a reliable CgA value.

Well-differentiated neuroendocrine tumours (NETs) are rare, slow-growing tumours. Chromogranin A (CgA) has replaced the 24-h urine collection of 5-hydroxyindoleacetic acid and has become the most important tumour marker in the monitoring of NET during and after treatment and is recommended by the European NeuroEndocrine Tumor Society (Korse *et al*, 2009; O'Toole *et al*, 2009). Chromogranin A is localised in the secretory granules of the neuroendocrine cells. Chromogranin A levels may also be elevated in other disease processes, such as prostate cancer, liver failure, cardiovascular diseases and renal failure (Tramonti *et al*, 2001; Modlin *et al*, 2010; Vezzosi *et al*, 2011). However, the use of proton pump inhibitors (PPIs) is the most important reason for an elevated CgA level, not caused by NET (Modlin *et al*, 2010). Thus far, the effect of discontinuation of PPI therapy was not investigated. This study examines whether discontinuation of PPI treatment, or replacement by H_2_-receptor antagonist (H2RA) therapy, decreases or even normalises the unexplained elevation of CgA levels in patients with well-differentiated NET.

## Materials and methods

For this prospective study, all patients diagnosed with well-differentiated NET visiting the Netherlands Cancer Institute between 2007 and 2009 with at least two available CgA measurements, were examined. Clinical data and medication were retrieved from medical records. Stage of disease was based on CT scans, or somatostatin receptor scintigraphy. Chromogranin A was determined with the CGA-RIA, a solid-phase, two-site immunoradiometric (CIS bio International, Gif-sur-Yvette, France) (Degorce *et al*, 1999; Korse *et al*, 2009). The reference value in our laboratory was established as <120 *μ*g l^−1^.

All patients with a normal CgA level during the study period were excluded from further examination. Patients with elevated CgA levels showing metastases or tumour progression were also excluded from further observation; these elevated CgA levels were attributed to tumour activity only. If patients showed elevated or increasing CgA levels that could not be attributed to the status of NET, the gastric acid inhibition treatment was registered. Those patients with unexpected elevated CgA levels and using PPI were asked to (temporarily) discontinue the PPI or to (temporarily) replace the PPI by H2RA treatment. After 2 weeks, the CgA level was measured again.

Wilcoxon paired tests and Mann–Whitney tests were applied to compare the CgA levels just before and 14 days after PPI discontinuation, and to compare change in CgA levels between patients who discontinued any acid suppressing therapy and patients who replaced PPI by H2RA. A change of >40% was considered as a significant change in the comparison of serial measurements (Dittadi *et al*, 2004).

## Results

Table 1 presents the characteristics of 196 patients at the time of the first CgA measurement: a total of 55 patients showed normal CgA levels and 122 patients had at least one elevated CgA level that was attributed to the status of the disease, according to imaging; these patients were not monitored longer for this study.

The remaining 19 patients had elevated CgA levels that could not be attributed to the disease status: 11 patients with no evidence of disease (NED) after surgery and eight patients with stable metastatic disease (SD) (Table 2). All these patients used PPI (omeprazole 20–40 mg daily) for dyspepsia or pyrosis, mainly prescribed by their general practitioner. In case of severe pyrosis, PPI was replaced by H2RA (ranitidine 300 mg daily, *n*=7). The remaining patients discontinued the acid inhibition completely (*n*=12). All 19 patients experienced no discomfort from this temporary cease of PPI use.

In the 11 patients with NED, the median CgA level decreased from 390 *μ*g l^–1^ during PPI treatment to 56 *μ*g l^–1^ after discontinuation of PPI (*P*=0.003). At that time, nine patients showed normal CgA levels, while in two patients (patients 6 and 7), the CgA levels decreased by 81% and 89%, respectively. The eight patients with SD according to imaging, showed unexpected increase of CgA levels. After discontinuation of PPI, the median CgA level decreased from 618 *μ*g l^–1^ during PPI use to 318 *μ*g l^–1^ (*P*=0.012). In two patients (patients 16 and 19), CgA levels even normalised.

All patients except two (patients 15 and 18) reached the critical difference of CgA change of >40% (33% and 35%, respectively). In 12 of the 19 patients with unexpected positive CgA levels who stopped all acid inhibition completely, CgA levels decreased by (median) 82% (range 33–89%). A similar trend was found in the seven patients who replaced PPI by H2RA: their CgA levels decreased by 77% (range 72–86%) (*P*=0.967).

## Discussion

In this study, we investigated the association of CgA levels and use of PPIs in patients treated for NET. High CgA levels were observed in 19 of the 196 (10%) patients, which could not be explained by tumour recurrence or progressive disease. All these patients were PPI users. An association between CgA levels and the use of PPI has already been described: a Dutch group published a cross-sectional study of 114 dyspeptic patients and showed that CgA levels were higher during long-term PPI treatment than during H2RA treatment, or no treatment (Sanduleanu *et al*, 1999). Giusti *et al* (2004) observed in six of seven patients a significant decrease of CgA levels after discontinuation of omeprazole. These studies suggested that decrease in acidity leads to release of gastrin. Elevated gastrin leads to hyperplasia of ECL cells and, subsequently, to an increase in CgA levels (Waldum *et al*, 1996; Sanduleanu *et al*, 1999). However, longitudinal studies investigating the effect of PPIs on CgA levels in patients with NET are lacking. In our group of NET patients, the elevated CgA levels significantly decreased, or even returned to normal, after discontinuation of gastric acid inhibition completely. A similar decrease was observed after replacement of PPI by H2RA.

A discontinuation of PPI of 2 weeks seemed a reasonable period in which to expect a decrease, similar to the report of Giusti *et al.* However, two patients (patients 6 and 7) with CgA levels >10 times the upper limit of normal showed an 81–89% decrease, but did not reach the normal range. Possibly, in patients with such extreme high values, a longer discontinuation is needed. It has also been suggested that cytochrome P450 2C19 (CYP2C19) might have a role in the metabolising of PPI. Even a five-fold higher exposure to PPIs has been observed in poor metabolisers (PMs) than in extensive metabolisers of CYP2C19 (Desta *et al*, 2002; Giusti *et al*, 2004). As the prevalence of PMs in Caucasian people is 1–3%, in some cases, the CgA could still be false positive after 2 weeks of discontinuation.

The two patients (patients 15 and 18) achieved a decrease <40%. Their CgA levels returned back to the precious levels (data not shown). Apparently, the CgA results were explained by both NET disease and PPI use. Since both the biological and assay variation of CgA, NET disease and PPI use might affect the CgA concentration, it is of crucial importance to stop PPI, to exclude the PPI influence on CgA levels. Patient 18 with liver metastases suffered also from renal failure (glomerular filtration rate=36 ml per minute per 1.73 m^2^), but even then the CgA level decreased with 35% (Modlin *et al*, 2010).

Of the 122 patients, 24 (20%) with high CgA levels used PPI. Examination of the medical reports showed that the concentration of CgA levels was fully attributed to the disease. In the group of 55 patients with normal CgA levels, 5 (9%) were PPI users. The duration of PPI use of these patients was unknown, but Sanduleanu *et al* (1999) suggested that the longer the use of PPI, the higher the CgA levels will be. In the future, all patients should discontinue PPI use in order to obtain a reliable CgA level, irrespective of tumour activity or duration of PPI use.

In our study population, there was no difference in CgA (percentage) decrease between patients who stopped acid inhibition completely and patients who replaced their PPI by H2RA. The use of H2RA did not affect the CgA levels, in contrast to PPI use. Proton pump inhibitor is a therapy that almost completely suppresses gastric acid. This is achieved by influencing the enzyme H^+^/K^+^-ATP-ase of the parietal cells (gastric proton pump). This acid suppression leads to an increase of gastrin, which is a trophic factor for hyperplasia of ECL cells. Apparently, the use of H2RA did not cause such hyperplasia of the ECL cells in this group of patients that it may lead to an increase of CgA. Although our H2RA group is relatively small, this finding confirms the results of Driman *et al* (1996) who reported increased parietal cell height, mass and number in omeprazole-treated patients compared with ranitidine-treated patients.

In conclusion, we recommend to cease or replace PPI by H2RA, to obtain a reliable value of CgA. A discontinuation of 2 weeks is usually sufficient.

## Figures and Tables

**Table 1:** Characteristics of the total study population

	***N*=196**
*Sex*	
Male	88 (45%)
Female	108 (55%)
	
*Age (years)*	
Median (range)	64 (18–89)
	
*Primary tumour site*	
Appendix	5 (3%)
Small bowel	86 (44%)
Large bowel	22 (11%)
Lung	21 (10%)
Pancreas	17 (9%)
Other	4 (2%)
Unknown	41 (21%)
	
*Metastases*	
No	57 (29%)
Yes	139 (71%)
	
*Carcinoid syndrome*	
No	66 (34%)
Yes	130 (66%)
	
*GFR*	
Normal	171 (87%)
Decreased	25 (13%)
	
*Number of CgA measurements*	
Median (range)	6 (2–27)
	
*CgA level*	
Normal	55 (28%)
Elevated (attributed to NET)	122 (62%)
Elevated (not attributed to NET)	19 (10%)
	
*PPI use*	
No	145 (74%)
Yes	51 (26%)

Abbreviations: CgA=chromogranin A; GFR=glomerular filtration rate; NET=neuroendocrine tumour; PPI=proton pump inhibitor.

**Table 2:** Change in CgA levels in 19 patients after discontinuation of PPIs treatment

**Patient**	**Sex, age (years)**	**Grade**	**Primary site**	**Status of disease**	**Carcinoid syndrome**	**GFR (ml per minute per 1.73 m^2^)**	**Replacement**	**CgA (*μ*g l^–1^) during PPI**	**CgA (*μ*g l^–1^) after PPI**	**CgA change (%)**
1	F, 63	G1	Appendix	NED	NA	85	H2RA	425	120	−72
2	F, 51	G2	Lung	NED	NA	86	H2RA	307	42	−86
3	F, 54	G2	Lung	NED	NA	76	Stop	178	51	−71
4	F, 42	G1	Lung	NED	NA	72	Stop	123	15	−88
5	M, 65	G1	Small bowel	NED	NA	95	Stop	266	40	−85
6	F, 77	G1	Small bowel	NED	NA	93	Stop	1310	245	−81
7	F, 61	G1	Small bowel	NED	NA	50	Stop	1610	181	−89
8	F, 65	G1	Small bowel	NED	NA	77	H2RA	206	48	−77
9	M, 76	G1	Small bowel	NED	NA	90	H2RA	390	65	−83
10	F, 67	G1	Pancreas	NED	NA	102	Stop	404	56	−86
11	M, 75	G1	Small bowel	NED	NA	59	Stop	576	101	−82
12	M, 68	G1	Pancreas	SD	Yes	82	H2RA	1367	332	−76
13	F, 67	G1	Small bowel	SD	Yes	78	H2RA	588	148	−75
14	F, 50	G1	Pancreas	SD	No	78	Stop	1330	305	−77
15	F, 66	G1	Small bowel	SD	No	60	Stop	646	436	−33
16	F, 56	G1	Unknown	SD	Yes	72	H2RA	235	38	−84
17	F, 60	G1	Unknown	SD	Yes	67	Stop	872	421	−52
18	M, 78	G1	Small bowel	SD	Yes	36	Stop	589	380	−35
19	M, 67	G1	Small bowel	SD	No	124	Stop	587	95	−84

Abbreviations: CgA=chromogranin A; F=female; GFR=glomerular filtration rate; H2RA=H_2_-receptor antagonist; M=male; NA=not applicable; NED=no evidence of disease; PPI=proton pump inhibitor; SD=stable disease.
